# Blood Gene Expression Predicts Bronchiolitis Obliterans Syndrome

**DOI:** 10.3389/fimmu.2017.01841

**Published:** 2018-01-11

**Authors:** Richard Danger, Pierre-Joseph Royer, Damien Reboulleau, Eugénie Durand, Jennifer Loy, Adrien Tissot, Philippe Lacoste, Antoine Roux, Martine Reynaud-Gaubert, Carine Gomez, Romain Kessler, Sacha Mussot, Claire Dromer, Olivier Brugière, Jean-François Mornex, Romain Guillemain, Marcel Dahan, Christiane Knoop, Karine Botturi, Aurore Foureau, Christophe Pison, Angela Koutsokera, Laurent P. Nicod, Sophie Brouard, Antoine Magnan, The COLT and SysCLAD Consortia, J. Jougon

**Affiliations:** ^1^Centre de Recherche en Transplantation et Immunologie UMR1064, INSERM, Université de Nantes, Nantes, France; ^2^Institut de Transplantation Urologie Néphrologie (ITUN), CHU Nantes, Nantes, France; ^3^UMR S 1087 CNRS UMR 6291, l’Institut du Thorax, Université de Nantes, CHU Nantes, Nantes, France; ^4^Pneumology, Adult Cystic Fibrosis Center and Lung Transplantation Department, Foch Hospital, Suresnes, France; ^5^Universite Versailles Saint-Quentin-en-Yvelines, UPRES EA220, Suresnes, France; ^6^Service de Pneumologie et Transplantation Pulmonaire, CHU Nord de Marseille, Aix-Marseille Université, Marseille, France; ^7^Groupe de Transplantation Pulmonaire des Hôpitaux universitaires de Strasbourg, Strasbourg, France; ^8^Hôpital Marie Lannelongue, Service de Chirurgie Thoracique, Vasculaire et Transplantation Cardiopulmonaire, Le Plessis Robinson, France; ^9^CHU de Bordeaux, Bordeaux, France; ^10^Hôpital Bichat, Service de Pneumologie et Transplantation Pulmonaire, Paris, France; ^11^Université de Lyon, INRA, UMR754, Lyon, Hospices Civils de Lyon, Lyon, France; ^12^Hôpital Européen George Pompidou, Paris, France; ^13^CHU de Toulouse, Toulouse, France; ^14^Hôpital Erasme, Bruxelles, Belgique; ^15^Clinique Universitaire Pneumologie, Pôle Thorax et Vaisseaux, CHU de Grenoble, Université de Grenoble, INSERM U1055, Grenoble, France; ^16^Service de Pneumologie, Centre Hospitalier Universitaire Vaudois (CHUV), Lausanne, Switzerland

**Keywords:** lung transplantation, bronchiolitis obliterans syndrome, gene expression, biomarkers, blood

## Abstract

Bronchiolitis obliterans syndrome (BOS), the main manifestation of chronic lung allograft dysfunction, leads to poor long-term survival after lung transplantation. Identifying predictors of BOS is essential to prevent the progression of dysfunction before irreversible damage occurs. By using a large set of 107 samples from lung recipients, we performed microarray gene expression profiling of whole blood to identify early biomarkers of BOS, including samples from 49 patients with stable function for at least 3 years, 32 samples collected at least 6 months before BOS diagnosis (prediction group), and 26 samples at or after BOS diagnosis (diagnosis group). An independent set from 25 lung recipients was used for validation by quantitative PCR (13 stables, 11 in the prediction group, and 8 in the diagnosis group). We identified 50 transcripts differentially expressed between stable and BOS recipients. Three genes, namely POU class 2 associating factor 1 (*POU2AF1*), T-cell leukemia/lymphoma protein 1A (*TCL1A*), and B cell lymphocyte kinase, were validated as predictive biomarkers of BOS more than 6 months before diagnosis, with areas under the curve of 0.83, 0.77, and 0.78 respectively. These genes allow stratification based on BOS risk (log-rank test *p* < 0.01) and are not associated with time posttransplantation. This is the first published large-scale gene expression analysis of blood after lung transplantation. The three-gene blood signature could provide clinicians with new tools to improve follow-up and adapt treatment of patients likely to develop BOS.

## Introduction

Chronic lung allograft dysfunction is the main limitation of long-term survival after lung transplantation. It manifests mainly by abnormal remodeling of the small airways, resulting in progressive airflow obstruction called bronchiolitis obliterans syndrome (BOS) (1–3). The prevalence of BOS is around 35% at 5 years. Its late diagnosis, based on the irreversible decline of lung function, attests to irreversible and advanced degradation of the allograft. Prognosis is poor, with a 4-year median survival after disease diagnosis (4). Thus, there is an urgent need to identify the predictors of BOS, which would allow proactive and targeted strategies to slow disease progression before irreversible degradation of the allograft occurs.

Bronchiolitis obliterans syndrome is likely to arise from repeated injuries from both alloimmune and non-alloimmune mechanisms, generating fibrosis and airway obstruction (5). Tracking these inflammation and fibrotic processes has been used to identify early signs of the disease, and bronchoalveolar lavage neutrophilia, levels of regulatory T cells, chemokines/cytokines, or matrix metalloproteases have been proposed as early biomarkers of BOS (6–10). More recently, expression profiling of lung biopsies pinpointed fibrosis-associated genes for the diagnosis or prediction of BOS (11). Yet, these invasive lung-centered approaches remain hampered by the accessibility to such samples and are therefore limited for routine monitoring of lung transplant recipients (LTRs). In blood, circulating fibrocytes and cytokine concentration have been proposed as predictors of BOS (12–16). However, these studies used a limited number of patients and have yet to be confirmed by follow-up studies. Consequently, none of these attempts have demonstrated sufficient feasibility and robustness to achieve clinical acceptance and potential routine use in the future.

Large-scale gene expression profiling of peripheral blood represents a promising tool to identify transcriptomic markers associated with the natural history of an allograft (17). The technique has been successfully tested for monitoring kidney (18), heart (19), and liver (20) transplant recipients. For the first time, we used this non-invasive approach to identify blood biomarkers of BOS in a large set of samples from LTRs. By using an independent set of patients, we were able to validate a transcriptomic signature that predicts the occurrence of BOS more than 6 months before the clinical manifestations.

## Materials and Methods

### Patients

Lung transplant recipients were recruited from September 2009 to October 2013 within the multicentre Cohort of Lung Transplantation (COLT) cohort (NCT00980967). The local ethical committee (*Comité de Protection des Personnes Ouest 1*-Tours, 2009-A00036-51) approved the study, and all participants provided written informed consent. Inclusion criterion was at least 3 years of follow-up unless the diagnosis of BOS was made before (Figure S1 in Supplementary Material).

The eligible patients (*n* = 688) were phenotyped by a blind adjudication committee as described previously (21, 22), based on pulmonary function tests and chest imaging according to ISHLT/ERS/ATS guidelines (3, 23). Assessed pulmonary tests were performed before the transplantation, the day of the transplantation, 1 and 6 months after the transplantation and every 6 months thereafter up to 3 years posttransplantation. We excluded 265 patients because of other phenotypes or confounding factors, and 338 stable patients and 85 BOS patients were identified. Stable patients displayed no signs of chronic dysfunction for at least 3 years after lung transplantation. Stable and BOS patients were then further selected to constitute homogenous groups based on sample availability, absence of concurrent infection or acute rejection within 1 month before or after blood collection, and the quality of RNA (RNA integrity number ≥6.5). Eighty-nine patients (49 STA and 40 BOS) were included in the identification set and 25 in the validation set (13 STA and 12 BOS).

### RNA Isolation

Peripheral blood samples were collected in PAXgene tubes (PreAnalytix, Qiagen, Hilden, Germany) and stored at −80°C. Total RNA was extracted using the PAXgene blood RNA system kit (Qiagen) with an on-column DNase digestion protocol according to the manufacturer’s instructions. Quantity and quality of total RNA were determined using a 2100 Bioanalyzer (Agilent Technologies, Palo Alto, CA, USA), and RNA samples with an RNA integrity number above 6.5 were selected for further analyses.

### Gene Expression Microarray Analysis

Total RNA (100 ng) were labeled using the Two-Color Agilent Low Input Quick Amp Labeling Kit and hybridized on SurePrint G3 Human Gene Expression v3 8 × 60K Microarrays following the manufacturer’s instructions (Agilent Technologies). Data extraction of median feature intensity was performed with Feature Extraction software v10.7 (Agilent Technologies). To remove signal intensity bias between each array, median feature intensities were normalized with the *lowess* (locally weighted scatter plot smoothing) method, then spots for which half the samples exhibited a signal less than the mean of all median signals were removed. Correction between two microarray hybridization batches was performed on the 28,867 remaining spots with the Combat algorithm (24) available through the R package *sva* (25). These normalized microarray data were deposited in the Gene Expression Ominbus database (accession number GSE94557). Mean expression levels for the spots targeting the same genes were assessed, resulting in 16,128 unique genes. In the BOS PRED group, the closest time point from transplantation was selected in cases of several samples per patient, so that no patient duplicate was included. Genes with low variation (i.e., variance <0.2), and thus considered as invariants, were excluded, resulting in 6,581 analyzed genes. For the identification of differential genes, linear modeling with empirical Bayes statistical procedure was performed, comparing the STA group and each group of interest, using the *limma* package in R. Genes with *p* < 5% and fold change (FC) >1.5 were considered as differentially expressed. The biological significance of selected genes was assessed using GOminer software. Only gene ontology (GO) categories enriched with a false discovery rate (FDR) <10% and with at least five represented genes were selected. The cell type source of differential genes was evaluated using the web tool Enrichr (26). Relative estimation of cell abundances using gene expression data was performed through the CIBERSORT software (27), and gene set enrichment analysis (GSEA) was performed to highlight relevant biological processes using curated gene sets from the Molecular Signatures Database (1,000 permutations and FDR threshold of 25%) (28).

### Quantitative PCR (qPCR) for Microarray Validation

After reverse transcription with Superscript III (Invitrogen, Carlsbad, CA, USA), real-time qPCR was performed on a Taqman StepOne plus real-time PCR system (Applied Biosystems, Foster City, CA, USA) using commercially available primers: *HPRT1* (Hs99999909_m1), β*2M* (Hs00984230_m1), *ACTB* (Hs99999903_m1), *CD19* (Hs99999192_m1), *TCL1A* (Hs00951350_m1), *IGLL5* (Hs04330879_u1), *POU2AF1* (Hs01573371_m1), and B cell lymphocyte kinase (*BLK*) (Hs01017452_m1). Samples were run in duplicate, and the geometric mean of quantification cycle values for *HPRT1*, β*2M*, and *ACTB* was used for normalization. Relative expression between a sample and a reference was calculated according to the 2^−ΔΔCt^ method.

### Gene Expression Data Sets

Gene expression values for the three genes of interest (*BLK, POU2AF1*, and *TCL1A*) from two public microarray data sets were collected from the Gene Expression Ominbus database: GSE38267 (29) and GSE28042 (30), corresponding to two studies analyzing blood gene expression in non-transplanted patients with terminal respiratory failure.

### Statistical Analysis

Non-parametric Kruskal–Wallis tests with Dunn’s *ad hoc* pairwise comparisons, Mann–Whitney tests, ROC curves, log-ranked survival analyses, and Fisher’s exact test for categorical variables were performed using GraphPad Prism v. 6 (GraphPad Software, La Jolla, CA, USA). Time-to-event analysis was performed using Cox proportional analysis between gene expression and time to BOS with the R *survival* package (*Coxph* function, R software v. 3.3.2).

## Results

### Lung Transplant Recipients

**Figure 1:**
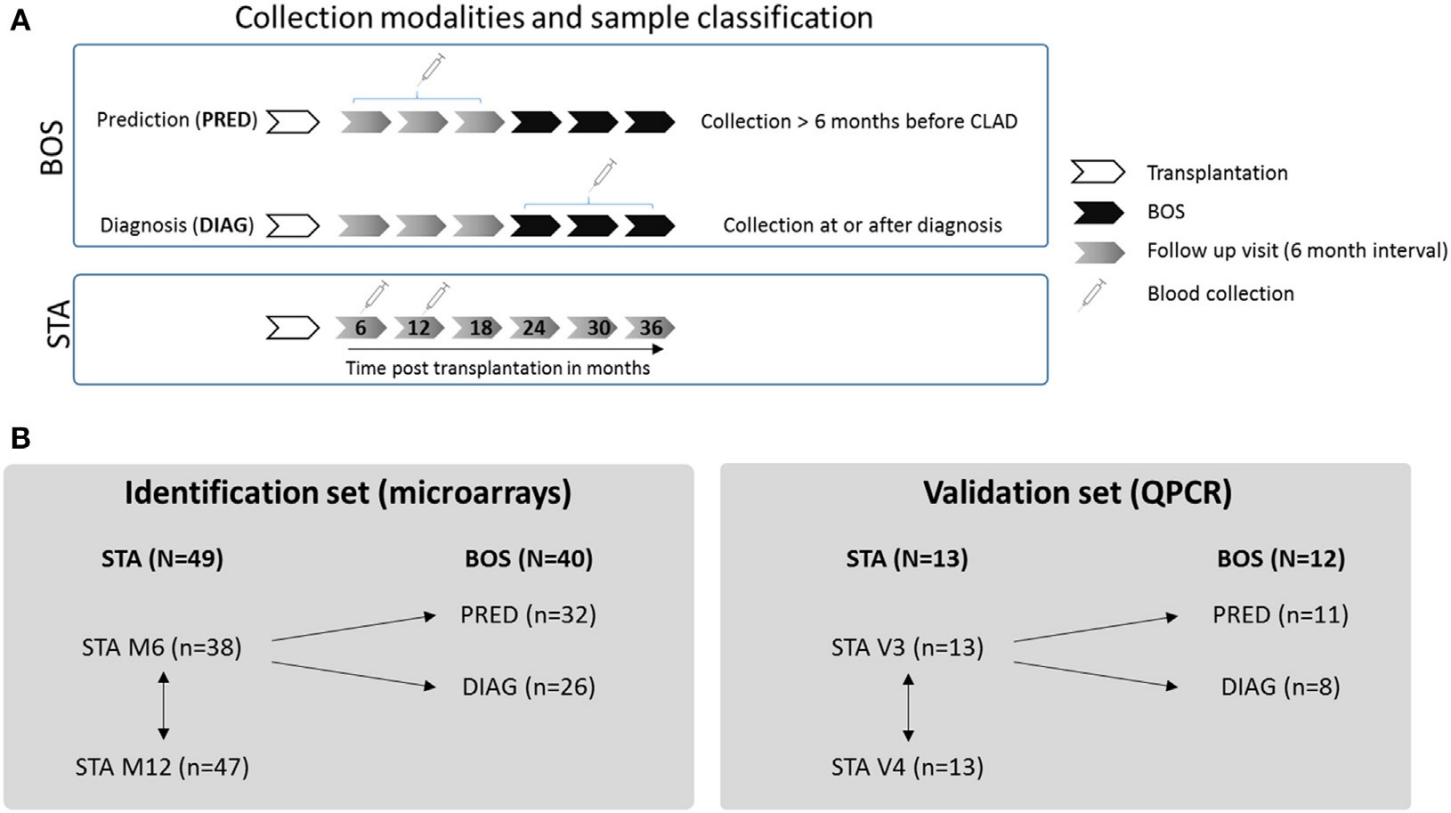
Experimental design. **(A)** Collection modalities and sample classification. For bronchiolitis obliterans syndrome (BOS) patients, two classes of samples were defined depending on the time between blood collection and BOS diagnosis: a prediction class (PRED) combining blood samples collected at least 6 months before BOS diagnosis and a diagnosis class (DIAG) combining blood samples collected at or up to 13 months after BOS diagnosis. For STA patients, samples collected 6 and 12 months after LT were used. **(B)** Strategy for gene expression analysis. In both identification and validation sets, STA samples collected 6 months posttransplantation were compared with PRED and DIAG class samples. No patient duplicate was included in these groups. For the STA group, comparison between gene expression at 6 and 12 months posttransplantation was performed to exclude irrelevant genes modulated with time.

Lung transplant recipients included in this study were recruited from the multicentre COLT cohort, allowing for longitudinal follow-up and 6-month interval biocollection from transplantation. On the basis of this longitudinal follow-up, we retrospectively defined two classes of BOS samples depending on the time between blood collection and BOS diagnosis (defined as the time point with a decline of ≥20% in FEV1 from baseline; Figure 1A). Blood samples collected at least 6 months before BOS diagnosis were included in the prediction class (PRED), and blood samples collected at the time or after BOS diagnosis (up to 13 months after initial diagnosis) were included in the diagnosis class (DIAG). For the group of patients with stable graft function (STA), blood was collected 6 and 12 months after transplantation, and a comparison of these two time points was performed to exclude irrelevant genes possibly altered during this interval after transplantation (Figure 1B). No patient duplicate was included within any classes.

Clinical parameters of patients in the identification and validation sets are presented in Table 1. LTR groups were homogeneous regarding age, sex, BMI, type of transplantation, induction treatment, smoking status, and infection and rejection events. A significant difference in azithromycin exposure was observed in the identification set between the DIAG and STA and PRED (76.9 vs. 22.4 and 37.5%, *p* < 0.0001). Predicted FEV1 was significantly lower in the DIAG in both cohorts (*p* < 0.05). Time of blood collection differed between the DIAG and PRED (*p* < 0.001).

**Table 1:** Patient characteristics.

	Identification set				Validation set			
		BOS (*N* = 40)				BOS (*N* = 12)		
	STA (*N* = 49)	DIAG (*N* = 26)	PRED (*N* = 32)		STA (*N* = 13)	DIAG (*N* = 8)	PRED (*N* = 11)	
Mean age at Tx (range)	40.2 (16–62)	40.6 (15–65)	43.3 (15–65)	ns	38.8 (23–62)	50.8 (29–63)	49.5 (27–63)	ns
Sex				ns				ns
M (%)	25 (51)	14 (53.8)	17 (53.1)		7 (53.8)	6 (75)	8 (72.7)	
F (%)	24 (49)	12 (46.2)	15 (46.9)		6 (46.2)	2 (25)	3 (27.3)	
BMI	20	20.5	21.2	ns	18.7	22.9	21.2	ns
Pathology leading to Tx				ns				ns
Emphysema/COPD, *n* (%)	11 (22.4)	8 (30.8)	9 (28.2)		3 (23)	3 (37.5)	5 (45.4)	
CF, *n* (%)	29 (59.2)	9 (30.6)	10 (31.3)		9 (69.2)	2 (25)	4 (36.4)	
PAH, *n* (%)	3 (6.1)	2 (7.7)	2 (6.3)		–	–	–	
IPF, *n* (%)	1 (2)	2 (7.7)	4 (12.5)		1 (7.8)	2 (25)	1 (9.1)	
Other, *n* (%)	5 (10.2)	5 (19.2)	7 (21.9)		–	1 (12.5)	1 (9.1)	
Type of Tx				ns				ns
Double lung, *n* (%)	41 (82.5)	19 (73.1)	24 (75)		11 (84.6)	7 (87.5)	10 (90.9)	
Single lung, *n* (%)	5 (12.5)	6 (23.1)	7 (21.9)		2 (15.4)	–	–	
Heart lung, *n* (%)	2 (2.5)	1 (3.8)	–		–	1 (12.5)	1 (9.1)	
Lobar, *n* (%)	1 (2.5)	–	1 (3.1)		–	–	–	
Induction				ns				ns
Basiliximab, *n* (%)	17 (34.7)	5 (19.2)	9 (28.1)		3 (23.1)	1 (12.5)	2 (18.2)	
Thymoglobulin, *n* (%)	17 (34.7)	12 (46.2)	16 (50)		7 (53.8)	1 (12.5)	2 (18.2)	
None, *n* (%)	15 (30.6)	9 (34.6)	7 (21.9)		3 (23.1)	6 (75)	7 (63.6)	
Immunosuppressives								
Steroids, *n* (%)	49 (100)	26 (100)	32 (100)		13 (100)	8 (100)	11 (100)	
Tacrolimus, *n* (%)	38 (79.6)	18 (69.2)	23 (71.9)		11 (84.6)	3 (37.5)	6 (54.5)	
Cyclosporin, *n* (%)	11 (22.4)	8 (30.8)	9 (28.1)		2 (15.4)	5 (62.5)	5 (45.4)	
MMF/MPA, *n* (%)	47 (95.9)	26 (100)	29 (90.1)		13 (100)	7 (87.5)	10 (90.1)	
Azithromycin	11 (22.4)	20 (76.9)^†^	12 (37.5)	*p* < 0.0001	6 (46.2)	7 (87.5)	6 (54.5)	
Acute cellular rejections				ns				ns
Ever A1 grade, *n* (%)	12 (24.5)	7 (26.9)	2 (6.25)		4 (30.7)	5 (62.5)	2 (18.2)	
Ever ≥A2 grade, *n* (%)	4 (8.2)	4 (15.4)	2 (6.25)		1 (7.7)	1 (12.5)	1 (9.1)	
Infections^a^								ns
Bacteria, *n* (%)	39 (79.6)	23 (88.5)	28 (87.5)		11 (84.6)	6 (75)	8 (72.7)	
Fungi, *n* (%)	28 (57.2)	21 (80.8)	21 (65.6)		6 (46.2)	4 (50)	7 (63.6)	
Virus, *n* (%)	19 (38.8)	16 (61.5)	14 (43.8)		3 (23)	4 (50)	3 (27.3)	
Collection time post-Tx (days ± SD)^b^	6 mo: 196 ± 31	642 ± 254^#^	210 ± 70^##^	*p* < 0.0001	6 mo: 185 ± 12	738 ± 86^#^	311 ± 39^##^	*p* < 0.0001
	12 mo: 375 ± 28				12 mo: 364 ± 9			
FEV1 (% predicted) at collection^b^	6 mo: 74.5 ± 19.5	50.7 ± 15.5^†^	72.5 ± 19.9	*p* < 0.0001	6 mo: 74.7 ± 17.1	57.7 ± 18.9^††^	83.4 ± 14.6	0.032
	12 mo: 80.6 ± 19.2				12 mo: 81.5 ± 18.4			
Smoking status				ns				ns
Non smoker	30	12	17		9	3	5	
Weaned	19	14	13		4	5	6	
Active smoker	0	0	2		0	0	0	

*Clinical parameters of the patients included in the identification and validation sets. The p values were calculated with the Fisher’s exact test for categorical variables and Kruskal–Wallis test with Dunn’s ad hoc pairwise comparisons for continuous variables*.

*^a^Number of patients with at least one event of infection during the follow-up*.

*^b^At 6 months and 12 months posttransplantation*.

*^#^p < 0.05 in DIAG vs. STA 6 mo and PRED*.

*^##^p < 0.05 in PRED vs. STA 12 mo*.

*^†^p < 0.0001 in DIAG vs. PRED and vs. STA*.

*^††^p < 005 in DIAG vs. STA 12 mo*.

### Identification of Gene Signatures Associated With BOS

**Figure 2:**
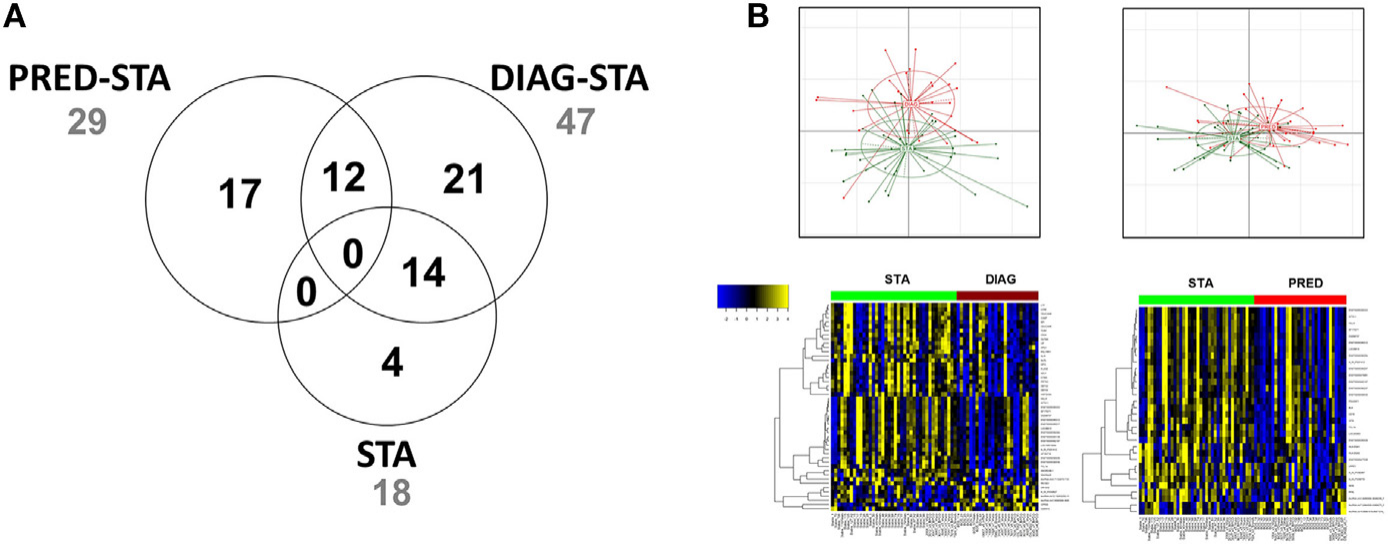
Differentially expressed genes. **(A)** Venn diagram of differentially expressed genes. Numbers of genes differentially expressed comparing STA vs. PRED, STA vs. DIAG, and STA at 6 and 12 months posttransplantation are indicated. **(B)** Principal component analysis and expression heat map (yellow and blue indicate high and low expression, respectively, and red and green indicate bronchiolitis obliterans syndrome and STA, respectively).

Gene expression profiling evidenced a total of 50 transcripts differentially expressed between STA and the 2 DIAG and PRED BOS groups, excluding differential transcripts between STA at the 2 time points (Figure 2; Table S1 in Supplementary Material). We identified 33 transcripts differentially expressed between STA and DIAG. While no GO term was significantly enriched, GSEA analysis highlighted 14 enriched gene sets (FDR < 25% and nominal enrichment <−2; Table S2 in Supplementary Material) among which 3 were related to B cell biology (31, 32) and one to upregulated genes in lung tissue of smokers with chronic obstructive pulmonary disease (COPD) (33).

Twenty-nine transcripts were differentially expressed between STA and PRED. According to the GO analysis, five genes participated to the enrichment of the immune system process (GO:0002376, FDR = 0.064): *CD19* (log_2_FC_PRED/STA_ = −0.60), the major histocompatibility complex class II DQα1 (*HLA-DQA1*, log_2_FC_PRED/STA_ = −0.67) and DQα2 (*HLA-DQA2*, log_2_FC_PRED/STA_ = −0.66), POU class 2 associating factor 1 (*POU2AF1*, log_2_FC_PRED/STA_ = −0.74), and Spi-B transcription factor (*SPIB*, log_2_FC_BOS/STA_ = −0.6). Analysis using the *Enrichr* tool stressed the enrichment of genes related to CD19^+^ B cells including *CD19, HLA-DQA1, POU2AF1*, B lymphoid tyrosine kinase (*BLK*, log_2_FC_PRED/STA_ = −0.62), and T-cell leukemia/lymphoma 1A (*TCL1A*, log_2_FC_PRED/STA_ = −0.80). Based on unsupervised hierarchical clustering of expressed genes, these genes clustered with known B cell-related genes such as *MS4A1* (membrane-spanning 4-domains, subfamily A, member 14 also called CD20 molecule), *BANK1* (B cell scaffold protein with ankyrin repeats 1), and *CD40*, reinforcing the potential association of B cell-related genes with prediction of BOS. Estimation of memory and plasma B cells relative abundances with gene expression using CIBERSORT did not highlighted significant alteration, while naive CD4^+^ T cells abundance was decreased in PRED compared to STA (Figure S2 in Supplementary Material). It is noteworthy that 14 transcripts, including *TCL1A*, immunoglobulin lambda-like polypeptide 5 gene (*IGLL5*), and various immunoglobulin lambda and kappa light chain variable regions, were associated with both the DIAG and the PRED.

While these results highlight genes differentially expressed between STA and each of the two BOS groups, BOS is a time-dependent phenomenon after lung transplantation. Thus, we reanalyzed differential genes from the STA and PRED comparison using a time-dependent analysis, the Cox proportional hazards test. According to the univariate analysis, all 29 differentially expressed genes displayed a significant association with BOS occurrence with time (*p* value of Wald test <0.05; Table S1 in Supplementary Material).

### Validation of *POU2AF1, TCL1A*, and *BLK* As Predictive Biomarkers of BOS

**Figure 3:**
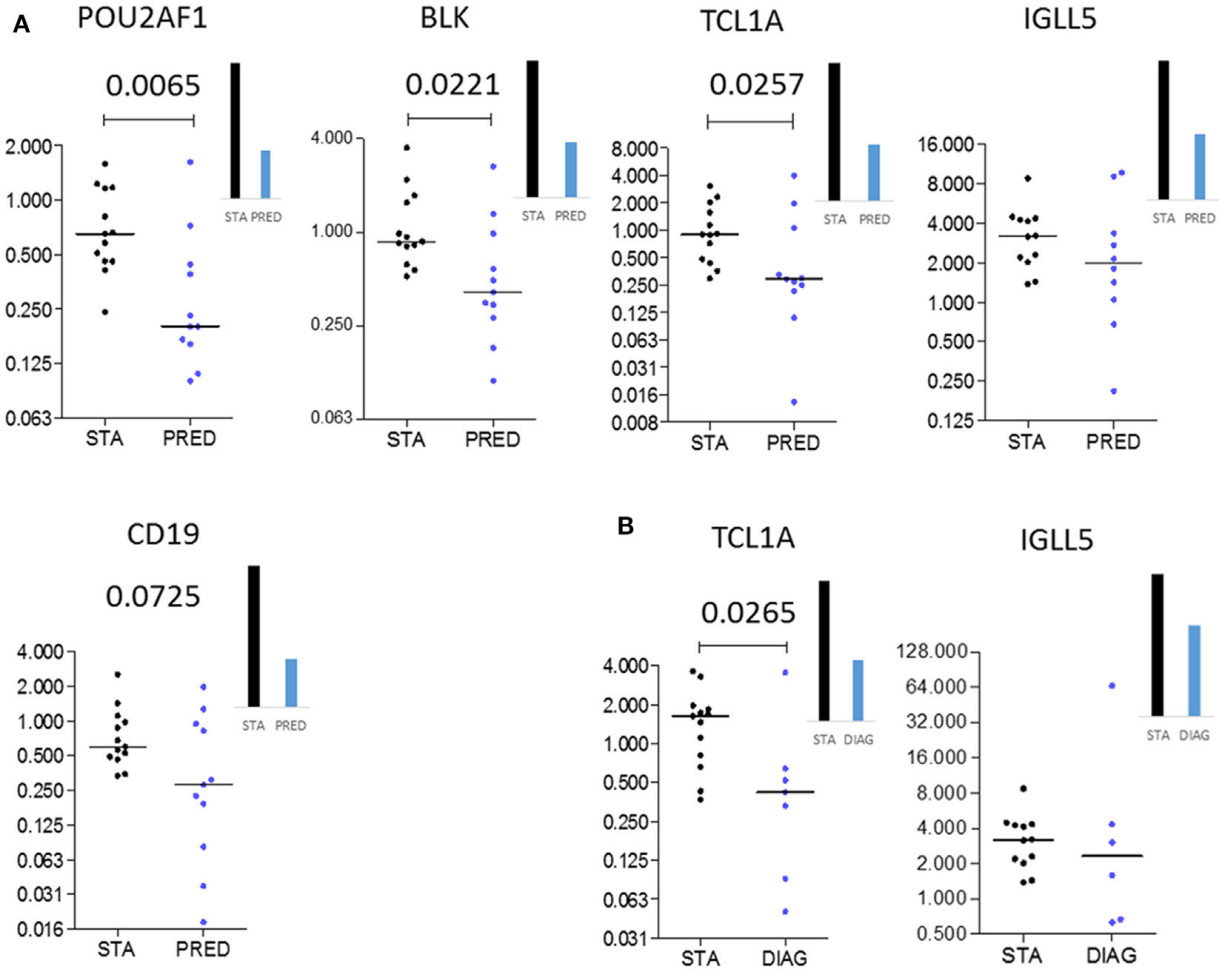
Independent validation. Microarray gene expression data (bar histograms) were validated by quantitative PCR in an independent set of patients (dot histograms) comparing STA and PRED **(A)** and STA and DIAG **(B)**. Mann–Whitney *p* values are indicated.

Among the differentially expressed genes between STA and PRED, five were selected on the basis of their *p* values, FC magnitude, and expression level in microarrays. These five genes were measured by qPCR in an independent set of 25 patients (13 STA and 12 BOS), with 11 and 8 samples in the PRED and DIAG, respectively (Table 2). Downregulation of 3 genes, *POU2AF1* (*p* = 0.0065), *TCL1A* (*p* = 0.0257), and *BLK* (*p* = 0.0221) in the PRED was validated by qPCR (Figure 3A). By contrast, the downregulation of *CD19* and *IGLL5* were not confirmed. Expression of *POU2AF1, TCL1A*, and *BLK* was constant in the STA between 6 and 12 months posttransplantation (Figure S3 in Supplementary Material) but was not significantly downregulated at the time of transplantation between STA and PRED (*n* = 6 and 9, respectively; Figure S4 in Supplementary Material). For diagnostic purposes, we confirmed the downregulation of *TCL1A* (*p* = 0.0265; Figure 3B).

**Table 2:** Microarray-identified genes selected for quantitative PCR (qPCR) validation.

Comparison	Symbol	Gene name	log_2_FC (BOS/STA)	Fold change (FC) [bronchiolitis obliterans syndrome (BOS)/STA]	*p* Value	Wald test
STA vs. PRED	*CD19*	CD19 molecule	−0.60	0.66	0.003	0.006
	BLK	B lymphoid tyrosine kinase	−0.62	0.65	0.000	0.001
	*POU2AF1*	POU class 2 associating factor 1	−0.74	0.60	0.001	0.003
	*TCL1A*	T-cell leukemia/lymphoma 1A	−0.80	0.57	0.003	0.003
	IGLL5	Immunoglobulin lambda-like polypeptide 5	−1.18	0.44	0.000	0.001

STA vs. DIAG	*IGLL5*	Immunoglobulin lambda-like polypeptide 5	−0.82	0.57	0.016	
	*TCL1A*	T-cell leukemia/lymphoma 1A	−0.79	0.58	0.006	

*Five genes were selected for qPCR validation based on their p values and FC magnitude. Gene name, function, p values, and FC values are presented*.

**Figure 4:**
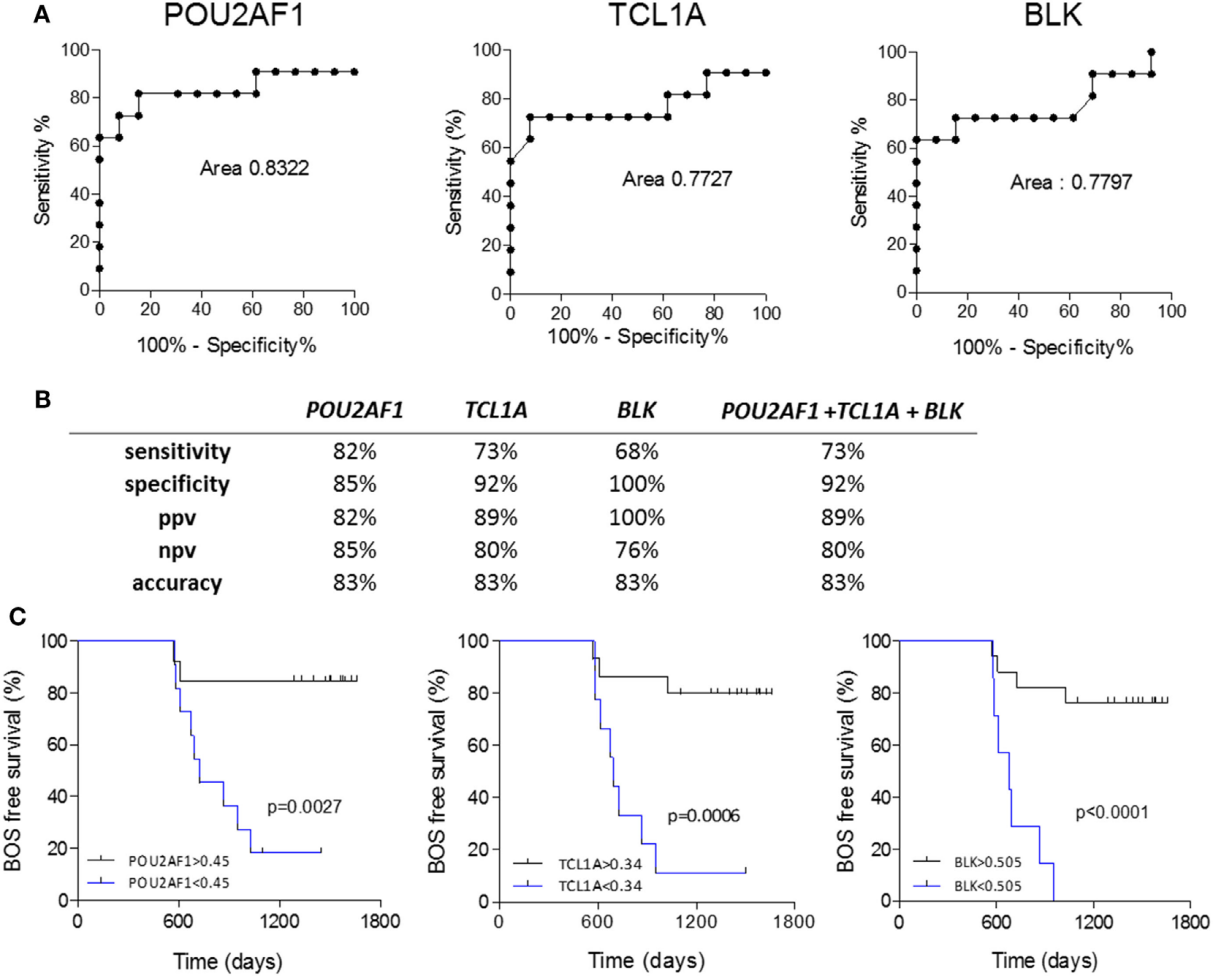
Performance of *POU2AF1, TCL1A*, and B cell lymphocyte kinase (*BLK*) in prediction of bronchiolitis obliterans syndrome (BOS). **(A)** ROC curves for *POU2AF1, TCL1A*, and *BLK* for the prediction of BOS are displayed. **(B)** Discriminative characteristics of the three genes or the sum of expression of the three genes. **(C)** Kaplan–Meier analysis of BOS-free survival categorized by best expression thresholds of discrimination in ROC curves. Log-ranked *p* values are indicated.

Because *POU2AF1, TCL1A*, and *BLK* were differentially expressed in the PRED, that is, more than 6 months before the clinical diagnosis of BOS, we evaluated the predictive performance of these three markers using ROC curve analysis. *POU2AF1* (AUC = 0.832, 95% CI = 0.638–1.026), *TCL1A* (AUC = 0.773, 95% CI = 0.553–0.993), and *BLK* (AUC = 0.780, 95% CI = 0.569–0.991) discriminated well between STA and BOS patients (Figure 4A). Global performances of the prediction presented in Figure 4 show an accuracy higher than 80% for the three markers. Due to the high level of correlation between the three genes, performance of the prediction was not improved by their combination (Figure S5 in Supplementary Material).

In the survival analysis, these three genes were highly associated with BOS occurrence with time in the discovery set (*BLK, p* = 0.0013; *POU2AF1, p* = 0.0028; and *TCL1A, p* = 0.0031; Table S1 in Supplementary Material). The association between BOS-free survival and *POU2AF1, TCL1A*, and *BLK* was assessed through Kaplan–Meier analyses. As shown in Figure 4C, expression levels of *POU2AF1, TCL1A*, and *BLK* under 0.45, 0.34, and 0.505, respectively (corresponding to best expression thresholds according to ROC curves) were associated with significant reduction of BOS-free survival after lung transplantation (*p* < 0.01). In this validation cohort, 3 of 8 recipients developing BOS had donor-specific antibody (DSA) at blood collection vs. none of the 13 STA (Fisher *p* = 0.031). Using univariate logistic analysis, DSA was not significantly associated with BOS.

### *POU2AF1, TCL1A*, and *BLK* Are Downregulated in Blood of Patients with End-Stage Chronic Respiratory Diseases

**Figure 5:**
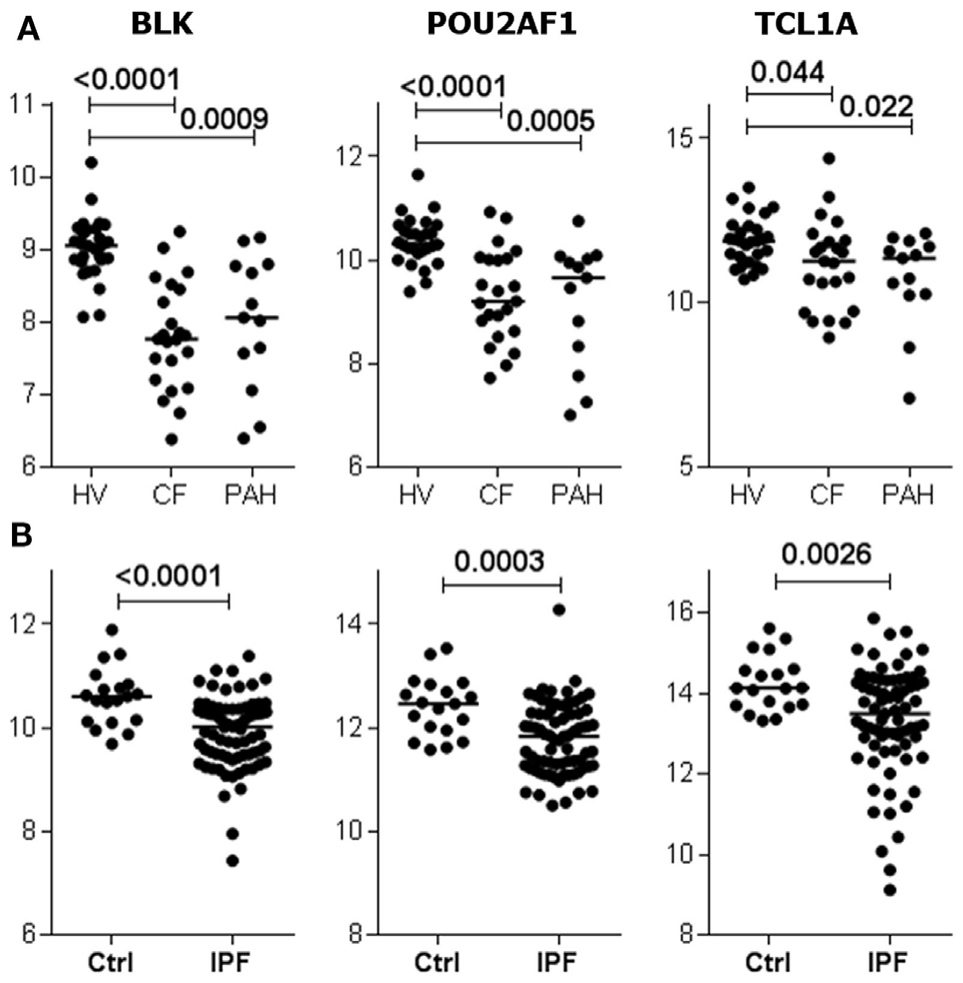
B cell lymphocyte kinase (*BLK*), *POU2AF1*, and *TCL1A* expression is downregulated by terminal respiratory failure. Expression of *BLK, POU2AF1*, and *TCL1A* in blood from non-transplanted patients with end-stage chronic respiratory diseases, displaying either cystic fibrosis (CF), pulmonary hypertension (PAH) **(A)**, or idiopathic pulmonary fibrosis (IPF) **(B)** compared to healthy volunteers (HVs) (29, 30). The *p* values from Kruskal–Wallis tests with Dunn’s *ad hoc* pairwise comparisons or Mann–Whitney tests are indicated.

Given the relationship between *POU2AF1, TCL1A*, and *BLK* expression and the development of BOS, we investigated the expression of these three genes in public data sets from blood of patients with other causes of respiratory failure (29, 30). The three genes were downregulated in the blood of non-transplanted patients with end-stage chronic respiratory diseases (cystic fibrosis, idiopathic pulmonary fibrosis, or pulmonary hypertension) compared to healthy volunteers (Figure 5).

## Discussion

Bronchiolitis obliterans syndrome is a major limitation of long-term survival after lung transplantation. Several previous attempts to identify early predictors of BOS remain limited by the absence of validation studies (6, 8–10, 12–14). Furthermore, they generally relied on invasive procedures incompatible with a routine clinical monitoring. We opted for a non-invasive large-scale molecular profiling approach to identify predictors of BOS in whole blood from 89 LTRs. A three-gene molecular signature differentiating BOS and STA was validated in an independent set of 25 patients. To the best of our knowledge, this is the first published study combining two independent cohorts for the identification and validation of predictors of BOS.

We showed that 29 and 33 transcripts were differentially expressed between STA and DIAG and STA and PRED. Twelve transcripts were common between both comparisons. This suggests a progression of the molecular profile during the development of the disease. Comparison of blood collected 6 and 12 months after transplantation for the STA group ruled out an early time-dependent alteration of gene expression. *POU2AF1, TCL1A*, and *BLK* were associated with the occurrence of BOS with time in a survival analysis and were validated as predictors of BOS more than 6 months before its diagnosis. The validation cohort used in our study revealed the strength of these biomarkers for BOS prediction. They displayed similar performances to predict BOS according to their correlation of expression, with AUC values reaching 0.83, 0.77, and 0.78, respectively, and exceeding the performance of biomarkers already published (14, 34, 35).

Recent literature highlights the correlation between *de novo* DSAs with the occurrence of chronic dysfunction (36–40). While in our validation cohort DSA was not significantly associated with BOS, an independent study is required to decipher whether these genes are associated with *de novo* DSA and BOS appearance and which parameter is the most predictive. Furthermore, our investigation focused on the BOS subtype, and further work will have to be conducted to identify predictors of the restrictive syndrome. Besides, stringent patient selection criteria were applied for a clear discrimination between groups, and all selected patients were not included in the analysis because of feasibility matters. This reduction in patient number may limit the generalizability of our results, whereas enough power was reached to highlight significantly statistical differences for the three genes. Although our predictions were confirmed on an independent set of patients, they will have to be validated on an external cohort as well.

Our primary objective was to identify a molecular biomarker for clinical monitoring in whole blood, which limits the interpretation of mechanistic investigations. Nevertheless, B cell signatures and the presence of B cells with suppressive properties are evidenced in other situations of transplantation and particularly in tolerance in kidney transplantation (41–43) and/or associated with long-term graft survival (44, 45). In a longitudinal study in lung transplantation, BOS appearance is associated with a lower level of CD24^hi^CD38^hi^IgD^hi^IgM^hi^ transitional B cells with a regulatory phenotype 18 months after lung transplantation compared to STA (46). We also observed that 12 and 13 transcripts related to immunoglobulins are downexpressed in BOS (PRED and DIAG, respectively) compared to STA, while gene expression of BAFF (B cell activating factor coded by *TNFSF13B*) and BAFF-R (BAFF receptor coded by *TNFRSF13C*), required for B cell survival and maturation, were not different between the three situations (data not shown). These data suggest a dysregulation of B cells in the BOS situation.

In addition, several genes merit further exploration to better understand the underlying mechanism behind BOS. By using GO analysis, we evidenced differentially expressed genes between STA *and* PRED associated with immune system process, such as *CD19, HLA-DQA1*, and *HLA-DQA2*. Interestingly, the gene enrichment and unsupervised hierarchical clustering analyses reinforce this association of the B cell cluster with the development of BOS. *POU2AF1* is a B cell transcriptional coactivator involved in B cell development and function (47). Yet, *POU2AF1* is expressed in T cells as well, and recent evidence revealed its role in Th1–Th2 polarization (48) and in the mounting of T-cell-dependent B cell responses (49). *BLK* is a member of the Src family of tyrosine kinases and encodes a non-receptor protein tyrosine kinase involved in the regulation of B cell receptor signaling (50). We found a downregulation of *TCL1A* in the BOS before disease onset. *TCL1A* is notably expressed by B and T lymphocytes, where it promotes cell proliferation and survival (51). Interestingly, in renal transplantation, *TCL1A* is downregulated at the time of acute allograft rejection (52), whereas it is overexpressed in operationally tolerant patients, an ideal situation where recipients are off of immunosuppression with a functioning allograft (53). The exact contribution of these genes in the development of BOS remains to be investigated. Some polymorphisms have been described for *BLK, POU2AF1*, and *TCL1A*. *BLK* polymorphism is a risk factor for developing autoimmune diseases (54, 55). Genetic variations leading to reduced BLK expression are associated with several autoimmune diseases *via* lowering the threshold for B cell activation (54). A *TCL1A* single-nucleotide polymorphism is associated with downstream expression of cytokines and chemokines and the nuclear factor-κB transcriptional activity resulting in the modulation of inflammation and immune response (56). *POU2AF1* polymorphism is associated with susceptibility to lymphoma (57), and mice deficient in *POU2AF1* exhibit reduced numbers of mature B cells and defective immune responses to antigens (58, 59). The fact that none of our patients evidenced clinical symptoms or biological associated modifications (such as decrease of total B cells), in addition with the absence of differential expression of these three genes at the time of transplantation do not suggest existence of such polymorphisms in our study. Intriguingly, however, the analysis of gene expression data from two independent studies (29, 30) revealed that *BLK, TCL1A*, and *POU2AF1* are downregulated in blood from patients with end-stage chronic respiratory diseases. GSEA analysis also highlighted the enrichment of a gene set related to COPD in lung from smokers in DIAG compared to STA, including the *SPIB* gene (33). Altogether, these results suggest common mechanisms between BOS and other chronic respiratory diseases, as previously noted (60), and support the need to further decipher the roles of *POU2AF1, BLK*, and *TCL1A* in the development of lung pathologies.

In conclusion, by using non-invasive whole blood profiling, we identified and validated *POU2AF1, TCL1A*, and *BLK* as three predictive biomarkers of BOS, more than 6 months before diagnosis. These genes allow stratification based on the BOS risk and could be easily monitored to provide clinicians with new tools to improve follow-up and adapt treatment of patient likely to develop BOS before clinical manifestations and allograft damage arise.

## Cohort of Lung Transplantation

**Cohort of Lung Transplantation, COLT** (associating surgeons, anesthetists-intensivists, physicians, research staff). **Bordeaux**: J. Jougon, J.-F. Velly, H. Rozé, E. Blanchard, C. Dromer. **Bruxelles**: M. Antoine, M. Cappello, R. Souilamas, M. Ruiz, Y. Sokolow, F. Vanden Eynden, G. Van Nooten, L. Barvais, J. Berré, S. Brimioulle, D. De Backer, J. Créteur, E. Engelman, I. Huybrechts, B. Ickx, T. J. C. Preiser, T. Tuna, L. Van Obberghe, N. Vancutsem, J.-L. Vincent, P. De Vuyst, I. Etienne, F. Féry, F. Jacobs, C. Knoop, J. L. Vachiéry, P. Van den Borne, I. Wellemans, G. Amand, L. Collignon, M. Giroux. **Grenoble**: E. Arnaud-Crozat, V. Bach, P.-Y. Brichon, P. Chaffanjon, O. Chavanon, A. de Lambert, J. P. Fleury, S. Guigard, K. Hireche, A. Pirvu, P. Porcu, R. Hacini, P. Albaladejo, C. Allègre, A. Bataillard, D. Bedague, E. Briot, M. Casez-Brasseur, D. Colas, G. Dessertaine, M. Durand, G. Francony, A. Hebrard, M. R. Marino, B. Oummahan, D. Protar, D. Rehm, S. Robin, M. Rossi-Blancher, P. Bedouch, A. Boignard, H. Bouvaist, A. Briault, B. Camara, S. Chanoine, M. Dubuc, S. Lantuéjoul, S. Quétant, J. Maurizi, P. Pavèse, C. Pison, C. Saint-Raymond, N. Wion, C. Chérion. **Lyon**: R. Grima, O. Jegaden, J.-M. Maury, F. Tronc, C. Flamens, S. Paulus, J. F. Mornex, F. Philit, A. Senechal, J.-C. Glérant, S. Turquier, D. Gamondes, L. Chalabresse, F. Thivolet-Bejui, C. Barnel, C. Dubois, A. Tiberghien. **Paris, Hôpital Européen Georges Pompidou**: F. Le Pimpec-Barthes, A. Bel, P. Mordant, P. Achouh, V. Boussaud, R. Guillemain, D. Méléard, M. O. Bricourt, B. Cholley, V. Pezella. **Marseille**: M. Adda, M. Badier, F. Bregeon, B. Coltey, X. B. D’Journo, S. Dizier, C. Doddoli, N. Dufeu, H. Dutau, J. M. Forel, J. Y. Gaubert, C. Gomez, M. Leone, A. Nieves, B. Orsini, L. Papazian L. C. Picard, M. Reynaud-Gaubert, A. Roch, J. M. Rolain, E. Sampol, V. Secq, P. Thomas, D. Trousse, M. Yahyaoui. **Nantes**: O. Baron, P. Lacoste, C. Perigaud, J. C. Roussel, I. Danner, A Haloun, A. Magnan, A. Tissot, T. Lepoivre, M. Treilhaud, K. Botturi-Cavaillès, S. Brouard, R. Danger, J. Loy, M. Morisset, M. Pain, S. Pares, D. Reboulleau, P. J. Royer, E. Durand, A. Foureau. **Le Plessis Robinson, Hôpital Marie Lannelongue**: Ph. Dartevelle, D. Fabre, E. Fadel, O. Mercier, S. Mussot, F. Stephan, P. Viard, J. Cerrina, P. Dorfmuller, S. Feuillet, M. Ghigna, Ph. Hervén, F. Le Roy Ladurie, J. Le Pavec, V. Thomas de Montpreville, L. Lamrani. **Paris, Hôpital Bichat**: Y. Castier, P. Cerceau, F. Francis, G. Lesèche, N. Allou, P. Augustin, S. Boudinet, M. Desmard, G. Dufour, P. Montravers, O. Brugière, G. Dauriat, G. Jébrak, H. Mal, A. Marceau, A.-C. Métivier, G. Thabut, B. Ait Ilalne. **Strasbourg**: P. Falcoz, G. Massard, N. Santelmo, G. Ajob, O. Collange, O. Helms, J. Hentz, A. Roche, B. Bakouboula, T. Degot, A. Dory, S. Hirschi, S. Ohlmann-Caillard, L. Kessler, R. Kessler, A. Schuller, K. Bennedif, S. Vargas. **Suresnes, Hôpital Foch**: P. Bonnette, A. Chapelier, P. Puyo, E. Sage, J. Bresson, V. Caille, C. Cerf, J. Devaquet, V. Dumans-Nizard, M. L. Felten, M. Fischler, A. G. Si Larbi, M. Leguen, L. Ley, N. Liu, G. Trebbia, S. De Miranda, B. Douvry, F. Gonin, D. Grenet, A. M. Hamid, H. Neveu, F. Parquin, C. Picard, A. Roux, M. Stern, F. Bouillioud, P. Cahen, M. Colombat, C. Dautricourt, M. Delahousse, B. D’Urso, J. Gravisse, A. Guth, S. Hillaire, P. Honderlick, M. Lequintrec, E. Longchampt, F. Mellot, A. Scherrer, L. Temagoult, L. Tricot, M. Vasse, C. Veyrie, L. Zemoura. **Toulouse**: J. Berjaud, L. Brouchet, M. Dahan, F. Le Balle, O. Mathe, H. Benahoua, A. Didier, A. L. Goin, M. Murris, L. Crognier, O. Fourcade.

**Swiss Transplant Cohort Study, STCS Genève-Lausanne.** T. Krueger, H. B. Ris, M. Gonzalez, J.-D. Aubert, L. P. Nicod, B. J. Marsland, T. C. Berutto, T. Rochat, P. Soccal, Ph. Jolliet, A. Koutsokera, C. Marcucci, O. Manuel, E. Bernasconi, M. Chollet, F. Gronchi, C. Courbon, Zurich S. Hillinger, I. Inci, P. Kestenholz, W. Weder, R. Schuepbach, M. Zalunardo, C. Benden, U. Buergi, L. C. Huber, B. Isenring, M. M. Schuurmans, A. Gaspert, D. Holzmann, N. Müller, C. Schmid, B. Vrugt, T. Rechsteiner.

**SME and Platforms. Biomax**, Germany: A. Fritz, D. Maier. **Finovatis**, Lyon, France: K. Desplanche, D. Koubi. **GATC**, Germany: F. Ernst, T. Paprotka, M. Schmitt, B. Wahl. **Novasdicovery**, Lyon, France: J.-P. Boissel, G. Olivera-Botello. **Prométhée Proteomics Platform**, Grenoble, France: C. Trocmé, B. Toussaint, S. Bourgoin-Voillard, M. Séve. **INSERM U823**, **Université Joseph Fourier**, Grenoble, France, M. Benmerad, V. Siroux, R. Slama. **European Institute for Systems Biology & Medicine**, Lyon, France: C. Auffray, D. Charron, J. Pellet, C. Pison.

## Ethics Statement

Lung transplant recipients were recruited from September 2009 to October 2013 within the multicentre COLT cohort (Cohort of Lung Transplantation; NCT00980967). The local ethical committee (Comité de Protection des Personnes Ouest 1-Tours, 2009-A00036-51) approved the study, and all participants provided written informed consent.

## Author Contributions

Conceived and designed the experiments/analyses: RD, PJR, SB, AM. Performed the experiments: RD, PJR, ED, JL. Analyzed the data: RD, PJR. Contributed samples/materials: DR, AT, PL, AR, MRG, CG, RK, SM, CD, OB, JFM, RG, MD, CK, KB, AF, CP, AK, LPN. Wrote the paper: RD, PJR, SB, AM. All authors approved the final version of the article.

## Conflict of Interest Statement

The authors declare that the research was conducted in the absence of any commercial or financial relationships that could be construed as a potential conflict of interest.
