# Supplementary material for: Blood Gene Expression Predicts Bronchiolitis Obliterans Syndrome
Source: Front Immunol. 2018 Jan 11;8:1841. doi: 10.3389/fimmu.2017.01841 (PMC5768645; doi:10.3389/fimmu.2017.01841)
Supplement: Supplementary file 1 [file Data_Sheet_1.docx]

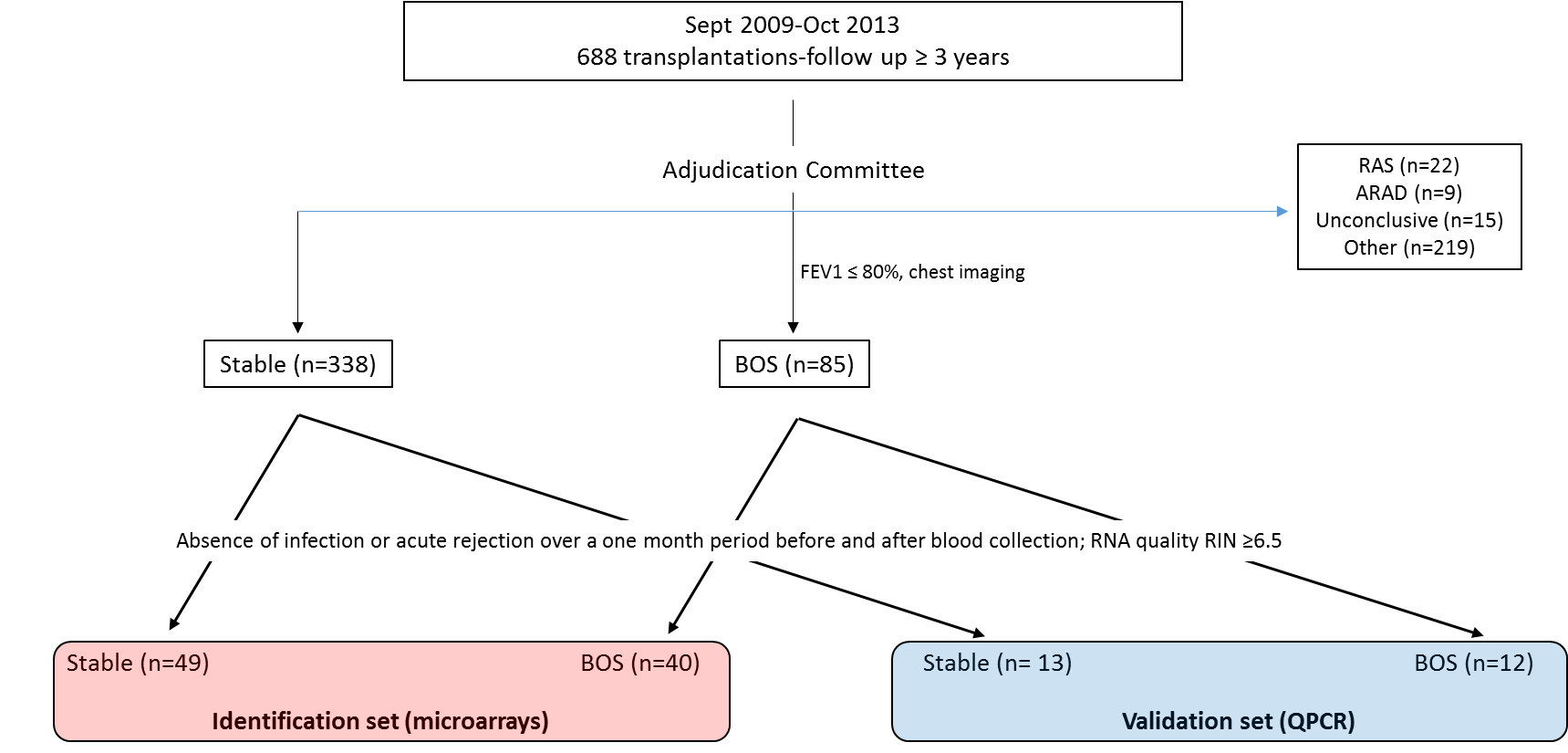


Supplementary Figure 1. Patient selection. Patients were recruited within the COLT cohort. Eligible patients were classified by an adjudication committee according to the ISHLT/ERS/ATS guidelines, and randomly allocated into the identification or the validation set.

**Supplementary Figure 2. Relative estimation of cell** **abundance for memory and plasma B cells and Naïve CD4^+^ Tcells using CIBERSORT analysis (**[**1**](#_ENREF_1)**)**

**
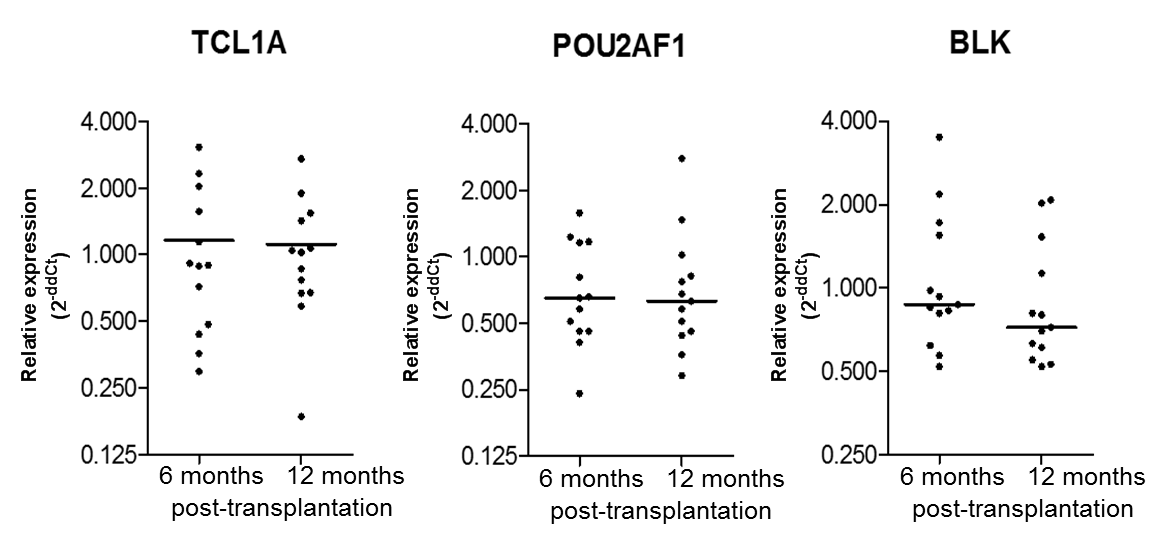
**

**Supplementary Figure 3. qPCR measure of *POU2AF1*, *TCL1A* and *BLK* in STA patients according time post-transplantation.** *POU2AF1*, *TCL1A* and *BLK* expression was determined by qPCR in STA patients from the validation set at 6 months and 12 months after transplantation.


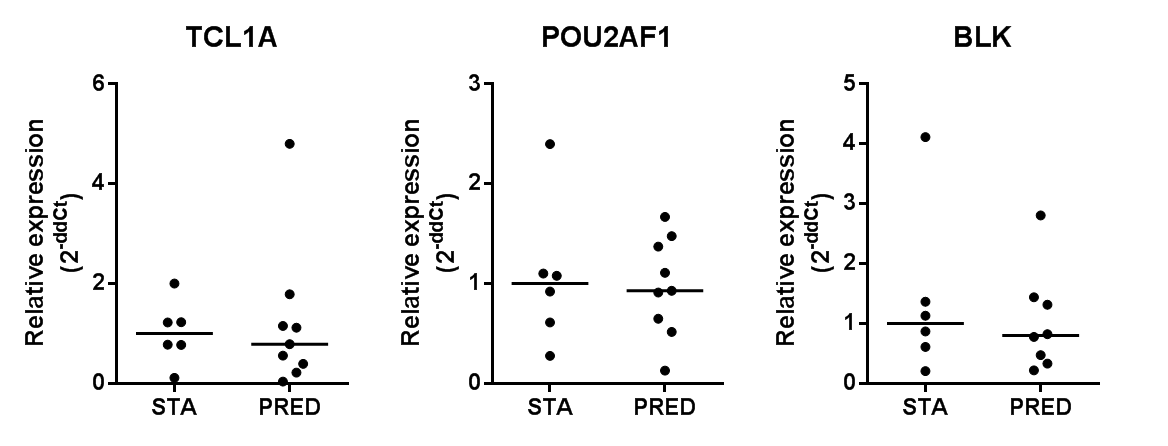


**Supplementary Figure 4. qPCR measure of *POU2AF1*, *TCL1A* and *BLK* in STA and PRED at transplantation time** (6 STA and 9 BOS)

**
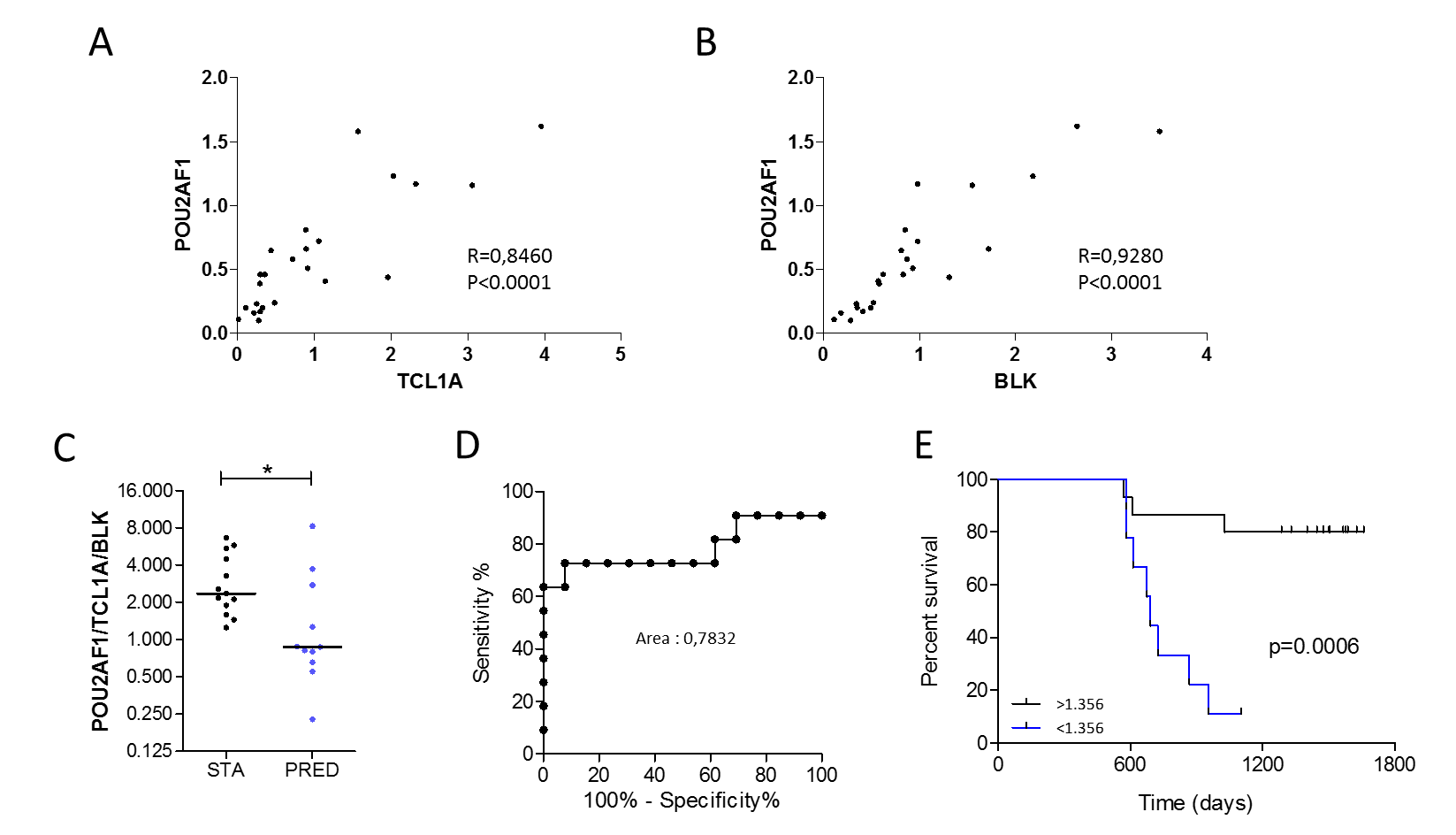
**

**Supplementary Figure 5. Combining *POU2AF1*, *TCL1A* and *BLK* does not improve the prediction of BOS.** Analysis of correlation between *POU2AF1* and *TCL1A* (**A**) and *POU2AF1* and *BLK* expression (**B**). Performance of the combination (sum of expression) was then investigated between STA and PRED class samples (**C**), ROC curve (**D**) and BOS-free survival curve (**D**).

**Supplementary Table 1. List of differentially expressed genes.** Genes in bold are common between STA-PRED and STA-DIAG comparison.

| **Comparison** | **Symbol** | **Gene name** | **log2FC (BOS/STA)** | **FC (BOS/STA)** | **P.Value** |
| --- | --- | --- | --- | --- | --- |
| **STA vs Diag** | AF194718 | clone 1120 immunoglobulin lambda light chain variable region mRNA, partial cds. [AF194718] | -0.60 | 0.66 | 0.003 |
|  | ALPL | alkaline phosphatase, liver/bone/kidney (ALPL), transcript variant 1, mRNA [NM_000478] | -0.68 | 0.62 | 0.004 |
|  | ARG1 | arginase 1, mRNA [NM_000045] | -0.81 | 0.57 | 0.015 |
|  | **BF175071** | cDNA, mRNA sequence [BF175071] | -0.90 | 0.53 | 0.002 |
|  | BPI | bactericidal/permeability-increasing protein (BPI), mRNA [NM_001725] | -0.61 | 0.66 | 0.029 |
|  | CDKN1C | cyclin-dependent kinase inhibitor 1C (p57, Kip2) (CDKN1C), transcript variant 1, mRNA [NM_000076] | 0.62 | 1.54 | 0.011 |
|  | **DQ098707** | isolate N1408L immunoglobulin lambda light chain variable region (IGLV2) mRNA, IGLV2-23*02 allele, partial cds. [DQ098707] | -0.71 | 0.61 | 0.004 |
|  | **ENST00000390294** | immunoglobulin lambda variable 1-47 [Source:HGNC Symbol;Acc:5880] [ENST00000390294] | -0.66 | 0.63 | 0.005 |
|  | **ENST00000390312** | immunoglobulin lambda variable 2-14 [Source:HGNC Symbol;Acc:5888] [ENST00000390312] | -0.74 | 0.60 | 0.003 |
|  | ENST00000390317 | immunoglobulin lambda variable 2-8 [Source:HGNC Symbol;Acc:5895] [ENST00000390317] | -0.64 | 0.64 | 0.005 |
|  | **ENST00000390323** | immunoglobulin lambda constant 2 (Kern-Oz- marker) [Source:HGNC Symbol;Acc:5856] [ENST00000390323] | -0.67 | 0.63 | 0.029 |
|  | ENST00000390556 | immunoglobulin heavy constant delta [Source:HGNC Symbol;Acc:5480] [ENST00000390556] | -0.59 | 0.67 | 0.001 |
|  | **ENST00000390559** | immunoglobulin heavy constant mu [Source:HGNC Symbol;Acc:HGNC:5541] | -0.70 | 0.62 | 0.008 |
|  | ENST00000483158 | immunoglobulin kappa variable 3-11 [Source:HGNC Symbol;Acc:5815] [ENST00000483158] | -0.64 | 0.64 | 0.004 |
|  | **ENST00000492167** | immunoglobulin kappa variable 3-20 [Source:HGNC Symbol;Acc:5817] [ENST00000492167] | -0.69 | 0.62 | 0.004 |
|  | GPR56 | G protein-coupled receptor 56 (GPR56), transcript variant 3, mRNA [NM_201525] | 0.61 | 1.53 | 0.017 |
|  | HIST2H3A | histone cluster 2, H3a (HIST2H3A), mRNA [NM_001005464] | -0.70 | 0.62 | 0.005 |
|  | **IGLL5** | immunoglobulin lambda-like polypeptide 5 (IGLL5), transcript variant 1, mRNA [NM_001178126] | -0.82 | 0.57 | 0.016 |
|  | lincRNA:chr12:132832202-132857482_F | lincRNA:chr12:132832202-132857482 forward strand | 0.67 | 1.59 | 0.028 |
|  | **lincRNA:chr3:36985666-36986208_F** | lincRNA:chr3:36985666-36986208 forward strand | 0.67 | 1.59 | 0.029 |
|  | lincRNA:chrX:71132475-71339425_R | lincRNA:chrX:71132475-71339425 reverse strand | -0.62 | 0.65 | 0.001 |
|  | LOC100510044 | ens\|immunoglobulin kappa variable 3-15 [Source:HGNC Symbol;Acc:5816] [ENST00000390252] | -0.61 | 0.65 | 0.015 |
|  | **LOC96610** | BMS1 homolog, ribosome assembly protein (yeast) pseudogene (LOC96610), non-coding RNA [NR_027293] | -0.76 | 0.59 | 0.003 |
|  | OR10H2 | olfactory receptor, family 10, subfamily H, member 2 (OR10H2), mRNA [NM_013939] | 0.59 | 1.51 | 0.022 |
|  | PGLYRP1 | peptidoglycan recognition protein 1 (PGLYRP1), mRNA [NM_005091] | -0.94 | 0.52 | 0.003 |
|  | PRTN3 | proteinase 3 (PRTN3), mRNA [NM_002777] | -0.67 | 0.63 | 0.006 |
|  | RN18S1 | RNA, 18S ribosomal 1 (RN18S1), ribosomal RNA [NR_003286] | -1.00 | 0.50 | 0.049 |
|  | **S77011** | {pseudogene} Ig V lambda IJ psi C lambda 6=Ig lambda chain surface Ig receptor VJC region {truncated light chain constant region} [human, B lymphoma cell line OCI LY8, mRNA Partial, 623 nt]. [S77011] | -0.79 | 0.58 | 0.011 |
|  | SLPI | secretory leukocyte peptidase inhibitor (SLPI), mRNA [NM_003064] | -0.68 | 0.62 | 0.009 |
|  | SNORA28 | small nucleolar RNA, H/ACA box 28 (SNORA28), small nucleolar RNA [NR_002964] | -0.76 | 0.59 | 0.012 |
|  | SNORD3B-1 | small nucleolar RNA, C/D box 3B-1 (SNORD3B-1), small nucleolar RNA [NR_003271] | -0.67 | 0.63 | 0.018 |
|  | **TCL1A** | T-cell leukemia/lymphoma 1A (TCL1A), transcript variant 1, mRNA [NM_021966] | -0.79 | 0.58 | 0.006 |
|  | TCN1 | transcobalamin I (vitamin B12 binding protein, R binder family) (TCN1), mRNA [NM_001062] | -0.74 | 0.60 | 0.008 |

| **Comparison** | **Symbol** | **Gene name** | **log2FC (BOS/STA)** | **FC (BOS/STA)** | **P.Value** | **WaldTest** |
| --- | --- | --- | --- | --- | --- | --- |
| **STA vs Pred** | **BF175071** | cDNA, mRNA sequence [BF175071] | -0.96 | 0.52 | 0.000 | 0.0023 |
|  | **DQ098707** | isolate N1408L immunoglobulin lambda light chain variable region (IGLV2) mRNA, IGLV2-23*02 allele, partial cds. [DQ098707] | -0.65 | 0.64 | 0.006 | 0.0142 |
|  | **ENST00000390294** | immunoglobulin lambda variable 1-47 [Source:HGNC Symbol;Acc:5880] [ENST00000390294] | -0.68 | 0.62 | 0.002 | 0.0095 |
|  | **ENST00000390312** | immunoglobulin lambda variable 2-14 [Source:HGNC Symbol;Acc:5888] [ENST00000390312] | -0.60 | 0.66 | 0.010 | 0.0161 |
|  | **ENST00000390323** | immunoglobulin lambda constant 2 (Kern-Oz- marker) [Source:HGNC Symbol;Acc:5856] [ENST00000390323] | -0.97 | 0.51 | 0.001 | 0.0035 |
|  | **ENST00000390559** | immunoglobulin heavy constant mu [Source:HGNC Symbol;Acc:5541] [ENST00000390559] | -0.68 | 0.63 | 0.006 | 0.0110 |
|  | **ENST00000492167** | immunoglobulin kappa variable 3-20 [Source:HGNC Symbol;Acc:5817] [ENST00000492167] | -0.73 | 0.60 | 0.001 | 0.0045 |
|  | **IGLL5** | immunoglobulin lambda-like polypeptide 5 (IGLL5), transcript variant 1, mRNA [NM_001178126] | -1.18 | 0.44 | 0.000 | 0.0010 |
|  | **lincRNA:chr3:36985666-36986208_F** | lincRNA:chr3:36985666-36986208 forward strand | -0.61 | 0.66 | 0.036 | 0.0481 |
|  | **LOC96610** | BMS1 homolog, ribosome assembly protein (yeast) pseudogene (LOC96610), non-coding RNA [NR_027293] | -0.61 | 0.65 | 0.011 | 0.0322 |
|  | **S77011** | {pseudogene} Ig V lambda IJ psi C lambda 6=Ig lambda chain surface Ig receptor VJC region {truncated light chain constant region} [human, B lymphoma cell line OCI LY8, mRNA Partial, 623 nt]. [S77011] | -0.93 | 0.52 | 0.001 | 0.0047 |
|  | **TCL1A** | T-cell leukemia/lymphoma 1A (TCL1A), transcript variant 1, mRNA [NM_021966] | -0.80 | 0.57 | 0.003 | 0.0031 |
|  | BLK | B lymphoid tyrosine kinase (BLK), mRNA [NM_001715] | -0.62 | 0.65 | 0.000 | 0.0013 |
|  | CD19 | CD19 molecule (CD19), transcript variant 2, mRNA [NM_001770] | -0.60 | 0.66 | 0.003 | 0.0060 |
|  | ENST00000390237 | immunoglobulin kappa constant [Source:HGNC Symbol;Acc:5716] [ENST00000390237] | -0.83 | 0.56 | 0.007 | 0.0168 |
|  | ENST00000390247 | immunoglobulin kappa variable 3-7 (non-functional) [Source:HGNC Symbol;Acc:5821] [ENST00000390247] | -0.61 | 0.66 | 0.006 | 0.0182 |
|  | ENST00000477036 | major histocompatibility complex, class II, DQ alpha 1 [Source:HGNC Symbol;Acc:4942] [ENST00000477036] | -0.89 | 0.54 | 0.008 | 0.0152 |
|  | ENST00000479981 | immunoglobulin kappa variable 1-16 [Source:HGNC Symbol;Acc:5732] [ENST00000479981] | -0.59 | 0.66 | 0.006 | 0.0160 |
|  | ENST00000498435 | immunoglobulin kappa variable 1-27 [Source:HGNC Symbol;Acc:5735] [ENST00000498435] | -0.61 | 0.66 | 0.008 | 0.0221 |
|  | HLA-DQA1 | major histocompatibility complex, class II, DQ alpha 1 (HLA-DQA1), mRNA [NM_002122] | -0.67 | 0.63 | 0.007 | 0.0122 |
|  | HLA-DQA2 | major histocompatibility complex, class II, DQ alpha 2 (HLA-DQA2), mRNA [NM_020056] | -0.66 | 0.63 | 0.002 | 0.0033 |
|  | lincRNA:chr10:80661019-80671219_F | lincRNA:chr10:80661019-80671219 forward strand | 0.59 | 1.50 | 0.034 | 0.0307 |
|  | lincRNA:chr7:33664500-33886275_R | lincRNA:chr7:33664500-33886275 reverse strand | 0.64 | 1.56 | 0.037 | 0.0332 |
|  | LOC283663 | hypothetical LOC283663 (LOC283663), non-coding RNA [NR_024433] | -0.93 | 0.52 | 0.000 | 0.0006 |
|  | LRRD1 | leucine-rich repeats and death domain containing 1 (LRRD1), mRNA [NM_001161528] | -0.76 | 0.59 | 0.013 | 0.0165 |
|  | NOG | noggin (NOG), mRNA [NM_005450] | -0.61 | 0.66 | 0.004 | 0.0126 |
|  | PFKL | phosphofructokinase, liver (PFKL), transcript variant 2, mRNA [NM_002626] | -0.65 | 0.64 | 0.014 | 0.0197 |
|  | POU2AF1 | POU class 2 associating factor 1 (POU2AF1), mRNA [NM_006235] | -0.74 | 0.60 | 0.001 | 0.0029 |
|  | SPIB | Spi-B transcription factor (Spi-1/PU.1 related) (SPIB), mRNA [NM_003121] | -0.60 | 0.66 | 0.001 | 0.0018 |

**Supplementary Table 2. List of enriched gene sets from the STA versus DIAG using GSEA analysis (**[**2**](#_ENREF_2)**)**

| **Gene set name** | **Description** | **Reference** | **Gene set size** | **Number of enriched genes** | **NES** | **NOM p-val** | **FDR q-val** | **Genes participationg to the gene set enrichment** |
| --- | --- | --- | --- | --- | --- | --- | --- | --- |
| ZHAN_MULTIPLE_MYELOMA_CD1_VS_CD2_DN | Genes down-regulated in CD-1 compared to CD-2 cluster of multiple myeloma samples. | ([3](#_ENREF_3)) | 15 | 11 | -2.12 | 0.0021 | 0.114 | LAPTM4B, ST6GAL1, , GPR160, PRDM5, PNOC, BLNK, CD27, C13orf15, PRKCA, NME4, STXBP6 |
| BASSO_B_LYMPHOCYTE_NETWORK | Genes which comprise the top 1% of highly interconnected genes that account for most of gene interactions in the reconstructed regulatory networks from expression profiles in B lymphocytes. | ([4](#_ENREF_4)) | 23 | 12 | -2.14 | 0.0020 | 0.128 | RPS26, SUN2, HHEX, BIN1, QPCT, ANXA6, ZYX, LGALS9, TNFRSF1B, HSPE1, FAIM3, NME1, CAMP, BLNK, HIST1H1E, P2RX5, HIST1H4B, IGLL1, BANK1, POU2AF1, MZB1, HIST1H4H, IL7R, CD24, MS4A1, HIST1H2BD, CD22, PTPRCAP, LTF, RPL29, , HIST1H2BD, HIST1H4E |
| BASSO_CD40_SIGNALING_DN | Gene down-regulated by CD40 signaling in Ramos cells (EBV negative Burkitt lymphoma). | ([4](#_ENREF_4)) | 27 | 13 | -2.12 | 0.0000 | 0.132 | ST6GAL1, BIK, NCF4, AQP3, BCL6, POU2AF1, PNOC, BLNK, ORAI2, CD27, LTB, SH2B2, CD19, ZNF85, LOC391322, ORAI2, PRTN3, PTPRCAP, PLP2, HLA-DQA1, ELANE, BCL2L1, HIST1H4J, HIST1H4C, HIST1H4D, HIST1H2BD, HIST1H4E, LCN2, TRIB3 |
| MULLIGHAN_NPM1_SIGNATURE_3_DN | Genes down-regulated in pediatric AML (acute myeloid leukemia) with mutated NPM1 (nucleophosmin) compared to the AML cases with intact NPM1 and MLL (lysine methyltransferase 2A). | ([5](#_ENREF_5)) | 48 | 9 | -2.08 | 0.0022 | 0.152 | FAM134B, SUN2, EPHX2, PDE4D, DEFA4, CD7, TCF7, RETN, HLA-DQA1, AP2A1, EEF2, PDIA4, BSG, MAF1, ACTN1, HIST1H4A, CD40LG, HIST1H4B, HIST2H3A, HIST1H4H, HIST1H4I, HIST1H2BG, RRAGD, CD7, LCN2, BPI, PRTN3 |
| ZAMORA_NOS2_TARGETS_DN | Down-regulated in hepatocytes upon expression of NOS2 (nitric oxide synthase 2). | ([6](#_ENREF_6)) | 30 | 12 | -2.04 | 0.0021 | 0.158 | EIF5A, VWF, JUP, IFNGR2, GNB2, F12, ALOX5AP, ATP6, HP, TCL1A, LBH, GNG7, ALPL, HIST1H4L, JUP, MMP9, P2RX5, TSPAN13, TMEM158, BANK1, TCN1, CEACAM6, RNASE3, RNASE2, IGLL1, ALOX5AP, ORAI2 |
| HADDAD_B_LYMPHOCYTE_PROGENITOR | Genes up-regulated in hematopoietic progenitor cells (HPC) of B lymphocyte lineage CD34+CD45RA+CD10+. | ([7](#_ENREF_7)) | 78 | 27 | -2.06 | 0.0041 | 0.158 | BLK, HIST1H2BI, LEF1, LILRA2, FAIM3, CD79B, VPREB3, FAM134B, UGCG, MAPK14, CEACAM3, DDAH2, HIST1H4K, ELANE, RPL18, EPHB4, ELANE, GSN, VWF, ANXA3, SLC11A1, RAB27A |
| PASINI_SUZ12_TARGETS_UP | Genes up-regulated in embryonic stem cells with defficient SUZ12 (polycomb repressive complex 2 subunit). | ([8](#_ENREF_8)) | 23 | 8 | -2.14 | 0.0000 | 0.159 | TCL1A, CENPM, HSD17B14, AGTRAP, TRIB3, UPP1, LY6G6E, AQP3, FBLN5, SLPI, HLA-DQA1, ELANE, CTSG |
| KOKKINAKIS_METHIONINE_DEPRIVATION_48HR_DN | Genes down-regulated in MEWO cells (melanoma) after 48h of methionine deprivation. | ([9](#_ENREF_9)) | 18 | 10 | -2.17 | 0.0020 | 0.159 | CDA, MYC, IFRD1, LEF1, BIRC5, PRKCA, TK1, UPP1, TYMS, MYL4, TFF3, MAL, HLA-DQA1 |
| YAGI_AML_WITH_T_8_21_TRANSLOCATION | Genes specifically expressed in samples from patients with pediatric AML (acute myeloid leukemia ) bearing t(8;21) translocation. | ([10](#_ENREF_10)) | 89 | 22 | -2.26 | 0.0000 | 0.160 | NUP214, CD74, JUP, TRAT1, CXCR5, PEBP1, CAPG, MPO, GGTLC1, HIST1H3D, CD19, BLNK, , CLEC1B |
| NING_CHRONIC_OBSTRUCTIVE_PULMONARY_DISEASE_UP | Up-regulated genes in lung tissue of smokers with chronic obstructive pulmonary disease (COPD) vs smokers without disease (GOLD-2 vs GOLD-0). | ([11](#_ENREF_11)) | 43 | 14 | -2.03 | 0.0000 | 0.161 | TLE2, VWF, RPL22, PGD, GNB2L1, LOC96610, SPIB, CLDN5, HLA-DOB, VAMP2, MYB, ACP5 |
| KEGG_SYSTEMIC_LUPUS_ERYTHEMATOSUS | Systemic lupus erythematosus -related KEGG pathway. |  | 67 | 27 | -2.04 | 0.0082 | 0.170 | HIST1H2BI, HIST2H2AC, HIST2H4B, HIST1H3F, CTSG, HLA-DOA, HIST1H3B, HLA-DQA2, PDLIM7, GNG7, CAMP |
| AMBROSINI_FLAVOPIRIDOL_TREATMENT_TP53 | Genes down-regulated by flavopiridol in the HCT116 cells (colon cancer) depending on their TP53 (tumor protein p53) status: wild-type vs loss of the gene's function. | ([12](#_ENREF_12)) | 25 | 10 | -2.02 | 0.0058 | 0.171 | FAM134B, BIN1, MICAL1, RRAGD, PASK, NEO1, RALGDS, FAIM3, GPA33, TBXA2R |
| VERHAAK_AML_WITH_NPM1_MUTATED_DN | Genes down-regulated in acute myeloid leukemia (AML) patients with mutated NPM1 (nucleophosmin). | ([13](#_ENREF_13)) | 106 | 29 | -1.98 | 0.0060 | 0.187 | TACSTD2, RETN, FAIM3, MPO, SPIB, UGCG, ABLIM1, TMEM204, ZDHHC18, AQP3 |
| SESTO_RESPONSE_TO_UV_C7 | Genes changed in primary keratinocytes by UVB irradiation. | ([14](#_ENREF_14)) | 26 | 8 | -2.00 | 0.0020 | 0.194 | GSN, TCN1, EPHX2, STMN1, TK1, PLP2, PGD, CKAP4, KIAA0319L |

**SI References**

1. Newman AM, Liu CL, Green MR, Gentles AJ, Feng W, Xu Y, et al. Robust enumeration of cell subsets from tissue expression profiles. Nature methods. 2015 May;12(5):453-7. PubMed PMID: 25822800. Pubmed Central PMCID: 4739640.

2. Subramanian A, Tamayo P, Mootha VK, Mukherjee S, Ebert BL, Gillette MA, et al. Gene set enrichment analysis: a knowledge-based approach for interpreting genome-wide expression profiles. Proceedings of the National Academy of Sciences of the United States of America. 2005 Oct 25;102(43):15545-50. PubMed PMID: 16199517. Pubmed Central PMCID: 1239896.

3. Zhan F, Huang Y, Colla S, Stewart JP, Hanamura I, Gupta S, et al. The molecular classification of multiple myeloma. Blood. 2006 Sep 15;108(6):2020-8. PubMed PMID: 16728703. Pubmed Central PMCID: 1895543.

4. Basso K, Margolin AA, Stolovitzky G, Klein U, Dalla-Favera R, Califano A. Reverse engineering of regulatory networks in human B cells. Nature genetics. 2005 Apr;37(4):382-90. PubMed PMID: 15778709.

5. Mullighan CG, Kennedy A, Zhou X, Radtke I, Phillips LA, Shurtleff SA, et al. Pediatric acute myeloid leukemia with NPM1 mutations is characterized by a gene expression profile with dysregulated HOX gene expression distinct from MLL-rearranged leukemias. Leukemia. 2007 Sep;21(9):2000-9. PubMed PMID: 17597811.

6. Zamora R, Vodovotz Y, Aulak KS, Kim PK, Kane JM, 3rd, Alarcon L, et al. A DNA microarray study of nitric oxide-induced genes in mouse hepatocytes: implications for hepatic heme oxygenase-1 expression in ischemia/reperfusion. Nitric oxide : biology and chemistry. 2002 Nov;7(3):165-86. PubMed PMID: 12381414.

7. Haddad R, Guardiola P, Izac B, Thibault C, Radich J, Delezoide AL, et al. Molecular characterization of early human T/NK and B-lymphoid progenitor cells in umbilical cord blood. Blood. 2004 Dec 15;104(13):3918-26. PubMed PMID: 15331438.

8. Pasini D, Bracken AP, Hansen JB, Capillo M, Helin K. The polycomb group protein Suz12 is required for embryonic stem cell differentiation. Molecular and cellular biology. 2007 May;27(10):3769-79. PubMed PMID: 17339329. Pubmed Central PMCID: 1899991.

9. Kokkinakis DM, Brickner AG, Kirkwood JM, Liu X, Goldwasser JE, Kastrama A, et al. Mitotic arrest, apoptosis, and sensitization to chemotherapy of melanomas by methionine deprivation stress. Molecular cancer research : MCR. 2006 Aug;4(8):575-89. PubMed PMID: 16908595.

10. Yagi T, Morimoto A, Eguchi M, Hibi S, Sako M, Ishii E, et al. Identification of a gene expression signature associated with pediatric AML prognosis. Blood. 2003 Sep 01;102(5):1849-56. PubMed PMID: 12738660.

11. Ning W, Li CJ, Kaminski N, Feghali-Bostwick CA, Alber SM, Di YP, et al. Comprehensive gene expression profiles reveal pathways related to the pathogenesis of chronic obstructive pulmonary disease. Proceedings of the National Academy of Sciences of the United States of America. 2004 Oct 12;101(41):14895-900. PubMed PMID: 15469929. Pubmed Central PMCID: 522001.

12. Ambrosini G, Seelman SL, Qin LX, Schwartz GK. The cyclin-dependent kinase inhibitor flavopiridol potentiates the effects of topoisomerase I poisons by suppressing Rad51 expression in a p53-dependent manner. Cancer research. 2008 Apr 01;68(7):2312-20. PubMed PMID: 18381438.

13. Verhaak RG, Goudswaard CS, van Putten W, Bijl MA, Sanders MA, Hugens W, et al. Mutations in nucleophosmin (NPM1) in acute myeloid leukemia (AML): association with other gene abnormalities and previously established gene expression signatures and their favorable prognostic significance. Blood. 2005 Dec 01;106(12):3747-54. PubMed PMID: 16109776.

14. Sesto A, Navarro M, Burslem F, Jorcano JL. Analysis of the ultraviolet B response in primary human keratinocytes using oligonucleotide microarrays. Proceedings of the National Academy of Sciences of the United States of America. 2002 Mar 05;99(5):2965-70. PubMed PMID: 11867738. Pubmed Central PMCID: 122456.

15. Gazda HT, Kho AT, Sanoudou D, Zaucha JM, Kohane IS, Sieff CA, et al. Defective ribosomal protein gene expression alters transcription, translation, apoptosis, and oncogenic pathways in Diamond-Blackfan anemia. Stem cells. 2006 Sep;24(9):2034-44. PubMed PMID: 16741228. Pubmed Central PMCID: 3372914.

16. Smid M, Wang Y, Zhang Y, Sieuwerts AM, Yu J, Klijn JG, et al. Subtypes of breast cancer show preferential site of relapse. Cancer research. 2008 May 01;68(9):3108-14. PubMed PMID: 18451135.
